# Editor of the American Journal of Dental Science

**Published:** 1869-10

**Authors:** W. H. Hoffman


### ARTICLE VII.

Lincolnton, N. C., Aug. 3d, 1869.

*Editor of the American Journal of Dental Science,*

DEAR SIR:—A case recently occurred with a lady patient of mine which is very singular.

Mrs.——, aged 30, was attacked with rubeola (measles) in December 1868, from which she suffered greatly, especially in the head.

She had worn a partial set of teeth (5 in number, 3 incisors and 2 bicuspids) for more than two and a half years with great comfort. After her recovery, to her great surprise and chagrin, she could not force her plate in her mouth, the plate being too large. Evidently the superior maxillary had undergone a change. The roof of the mouth becoming more flat, as the plate would rock upon reaching the hard palate of the mouth.

Will you be so kind as to inform me of the cause, or in any manner account for this singular or abnormal condition.

I am with high regard,

W. H. HOFFMAN.

*Remarks.* The probability is that in the above case the mucous membrane of the mouth was the part alone in which the change occurred, without there was present one or more cases of alveolar abscess about the teeth remaining in the jaw. We cannot conceive how the attack of rubeola could have had any effect whatever upon the bony structure, but that it should upon the mucous membrane is much more probable.—(ED).

